# Ameliorative Effect of *Annona muricata* (Graviola) Extract on Hyperglycemia Induced Hepatic Damage in Type 2 Diabetic Mice

**DOI:** 10.3390/antiox10101546

**Published:** 2021-09-29

**Authors:** Yiseul Son, Heaji Lee, Su-Young Son, Choong-Hwan Lee, Sun-Yeou Kim, Yunsook Lim

**Affiliations:** 1 Department of Food and Nutrition, Kyung Hee University, 26 Kyung Hee-Daero, Seoul 02447, Korea; sondltmf55@khu.ac.kr (Y.S.); ji3743@khu.ac.kr (H.L.); 2 Department of Bioscience and Biotechnology, Konkuk University, Seoul 05029, Korea; syson119@konkuk.ac.kr (S.-Y.S.); chlee123@konkuk.ac.kr (C.-H.L.); 3 Gachon Institute of Pharmaceutical Science, Gachon University, #191, Hambakmoero, Incheon 21936, Korea; sunnykim@gachon.ac.kr

**Keywords:** type 2 diabetes mellitus (T2DM), hepatic lipid steatosis, non-alcoholic fatty liver disease (NAFLD), lipophagy, autophagy, AMPK/Akt-mTOR signaling, *Annona muricata*, graviola

## Abstract

*Annona muricata* (AM) is evergreen plant of the *Annonaceae* family and known to have anticancer and antidiabetic effects. However, anti-diabetic mechanisms of AM extracts (AME) associated with hepatic glucose regulation and lipid metabolism remain unclear. In this study, we investigated the protective effect of AME extracted on hepatic damage in diabetic mice. Diabetes was induced by a high-fat diet with two-times streptozotocin (STZ) injection (60 mg/kg BW) in C57BL/6 male mice. The diabetic mice were daily administered with AME (50 or 100 mg/kg BW) by gavage for 9 weeks. Biomarkers related to energy metabolism and insulin signaling were examined to identify the effect of AME on hyperglycemia induced hepatic damage. AME supplementation reduced levels of FBG, HbA1c, HOMA-IR and hepatic lipid profiles as well as enhanced insulin signaling by increased the protein levels of IRS-1 accompanied GLUT2 in diabetic mice. Especially low dose of AME showed the beneficial effect of reducing oxidative stress (4-HNE, protein carbonyls, Nrf2, NQO1) and improved hepatic morphology demonstrated by lipid droplets along with upregulation of lipophagy (pAMPK, p-mTOR/mTOR, LC3-2/LC3-1) in diabetic mice. Moreover, AME supplementation ameliorated hepatic lipid metabolism (FAS, SREBP1c, C/EBPα, PPARγ, CPT1A, PPARα) and energy metabolism (pAMPK, PGC1α) in diabetic mice. Taken together, this study suggested that AME could be helpful to prevent hepatic abnormality by regulation of insulin signaling associated with energy metabolism and autophagy in diabetes.

## 1. Introduction

Type 2 diabetes mellitus (T2DM), called non-insulin dependent diabetes, is a chronic disease that occurs when the body cannot effectively use the insulin it produces [1]. It is estimated that in 2018, the number of people with type 2 diabetes was more than 500 million cases worldwide [2]. Common symptoms of T2DM are hyperglycemia and dyslipidemia, which cause multi-organ damage in the body [3]. The liver is an insulin-sensitive tissue and is highly susceptible to hyperglycemic-induced oxidative stress that can cause tissue damage [4]. According to previous studies, T2DM was known as a risk factor for several liver diseases such as non-alcoholic fatty liver disease (NAFLD), hepatitis, fibrosis, cirrhosis and hepatocellular carcinoma (HCC) [5,6,7,8].

Insulin resistance in T2DM patients leads to an imbalance in hepatic lipid metabolism. Excess free fatty acids (FFAs) in diabetes exacerbate positive feedback that induces further insulin resistance [9]. The key factor in insulin-regulated lipid homeostasis is the sterol response element binding protein 1c (SREBP1c), which regulates the expression of genes involved in the synthesis and absorption of fatty acids, triglycerides (TG), cholesterol and phospholipids. The stimulation of SREBP1c due to insulin resistance and hyperglycemia increases the rate of de novo fatty acid synthesis through activation of lipogenic enzymes such as fatty acid synthase (FAS) and acetyl-CoA carboxylase (ACC) [10]. In addition, transcription factors such as CCAAT-enhancer-binding proteins-α (C/EBPα) and peroxisome proliferator-activated receptor-γ (PPARγ) also increase hepatic fat accumulation. On the other hand, inhibition of peroxisome proliferator-activated receptor-α (PPARα) and carnitine palmitoyl-transferase 1 (CPT1) reduces β-oxidation of fatty acids [11]. This imbalance between lipogenesis factors and β-oxidation factors causes hyperlipidemia followed by accumulation of TG in hepatocytes and develops NAFLD [12].

One of the major mechanisms in hepatic lipid dysregulation such as NAFLD and hepatic steatosis is autophagy. The liver converts and stores TG into lipid droplets (LDs) to defend hepatic tissue against lipid toxicity during diabetic-hyperlipidemia [13]. However, in order to prevent excessive accumulation of LDs, fat decomposition is activated to maintain the size of the LD [14]. This mechanism for balancing liver fat stores is an autophagy pathway called lipophagy [15]. LC3B-II, a major marker of the autophagy pathway, was significantly reduced in insulin-resistant mice [16]. The result suggests that augmentation of insulin resistance induces NAFLD by reducing lipophagy in T2DM. AMP-activated protein kinase/mammalian target of rapamycin (AMPK/mTOR) signaling pathway is one of the major regulatory pathways of autophagy [17]. When AMPK is activated due to decreases in intracellular ATP levels, it inhibits the activity of mTOR. Whereas phosphorylated mTOR (p-mTOR) inhibits autophagy, negative regulation of mTOR promotes autophagy [18]. In diabetic conditions, an increased level in mTOR accompanied by a decreased level in AMPK caused by hyperglycemia plays as negative feedback of autophagy.

In addition, hepatic lipid overload induces the overproduction of oxidants which contributes to progression of hepatic damage in T2DM. The nuclear factor erythrocyte-related factor 2 (Nrf2) transcription factor is essential for maintaining cellular homeostasis in the presence of reactive oxygen species (ROS) or reactive nitrogen species (RNS) produced by internal metabolism or external exposure [19]. The active form of Nrf2 transcriptionally controls several cytoprotective genes. It is also involved in the direct reduction of ROS and RNS by promoting the expression of inhibitory factors [20]. Under the oxidative stress conditions, pathological mechanism affects several ROS-generating mechanisms which influence lipid metabolism and insulin signaling in T2DM [21].

*Annona muricata*, called graviola is an evergreen plant of the *Annonaceae* family, mainly distributed in tropical and subtropical parts of the world. The major bioactive components of graviola is acetogenins, rutin, quercetin, kaempferol which have been shown to have anti-cancer and anti-arthritis, liver protective effect in previous studies [22,23,24]. In particular, graviola has been traditionally used for treatment of diabetes. Several studies reported that extract of graviola leaves improved blood sugar and lipid levels in diabetic mice. They also showed antioxidant effect and protective effect on pancreatic β cells [25,26,27]. However, few studies have shown anti-diabetic mechanism of graviola related hepatic glucose regulating, lipid metabolism and molecular signaling. Accordingly, this study aims to investigate the mechanisms of the antidiabetic effect of graviola on hyperglycemia induced hepatic damage in T2DM mice.

## 2. Materials and Methods

### 2.1. Preparation of Annona Muricata Extracts (AME)

*Annona muricata* leaves were grown in Indonesia and purchased through DS International (Jeungpyeong-gun, Chungcheongbuk-do, Korea) in dried form. That was extracted twice in 50% ethanol at room temperature. The extract was prepared in powder form through solvent evaporation, freeze drying and powdering. In this process, 6.7 g of extract powder was taken from 100 g of dried leaves. The extraction yield was about 6.73%. Before the experiment, it was dissolved in distilled water at a suitable concentration for the experimental design.

### 2.2. UHPLC Analyses

The dried AME were re-dissolved in 80% MeOH for ultra-high-performance liquid chromatography-linear trap quadrupole-orbitrap-tandem mass spectrometry (UHPLC-LTQ-Orbitrap- MS/MS) analysis to determine the components of AME.

UHPLC system consisting of a Vanquish binary pump H system (Thermo Fisher Scientific Co., Ltd., Waltham, MA, USA), autosampler, column compartment, and a detector. Sample separation was performed on a Phenomenex KINETEX^®^ C18 column (100 mm × 2.1 mm, 1.7 μm) and the column temperature was set to 40 °C, and the flow rate was 0.3 mL/min. The MS data were collected in the range of 100–1500 *m/z* (under both negative- and positiveion modes) using an Orbitrap Velos ProTM system, which was combined with an ion-trap mass spectrometer (Thermo Fisher Scientific) coupled with a HESI-II probe.

### 2.3. Total Polyphenol Contents

The Folin–Ciocalteu test was used to measure total polyphenol contents of AM extracts (AME). The crude sample was prepared to yield a concentration of 1 mg/mL. About 500 µL of the extract (1 mg/mL) was combined and mixed with 0.5 mL of the Folin–Ciocalteu reagent in the test tube. The liquid mixture was allowed to stand for 3 min at a room temperature. The mixture was then added about 1.5 mL of sodium carbonate (Na_2_CO_3_), and the test tube was shaken gently to mix them. After 60 minutes, the absorbance of the mixture was measured at 725 nm.

### 2.4. Animals and Diabetes Induction

Four-week-old C57BL/6J male mice (*n* = 68) were obtained from Dae-han Bio Link (DBL; Eumseong-gun, Chungcheongbuk-do, Korea). All mice were caged in two or three per cages under regulated conditions 12-h light-dark cycles, constant temperature (22 ± 1 °C) and humidity (50 ± 5%). After a week with acclimation, all mice were randomly divided into two groups: a non-diabetic control group (NC; *n* = 11) and a diabetic group (DM; *n* = 57). The DM group was fed with 60% kcal high-fat diet (D12492; Research Diets, New Brunswick, NJ, USA), while the NC group was fed with 10% kcal control diet (D12450J; matching sucrose to D12492, Research Diets, New Brunswick, NJ, USA). The diet and water were provided *ad libitum*. Four weeks later, the DM group induced type 2 diabetes by intraperitoneal (i.p.) injection of streptozotocin (STZ; 60 mg/kg body weight, Sigma Aldrich) with citrate buffer (pH 4.5) at once a week for a second consecutive week. The NC group was injected with citrate buffer under the same conditions as the DM group. Every week after the last injection, 8 h-fasting blood glucose (FBG) was measured from the mice tail vein using One-touch select glucometer (LifeScan Inc., Milpitas, CA, USA). Mice with FBG levels higher than 250 mg/dl at least twice in four weeks were considered as diabetic and used for the experiment [28]. All animal experimental procedures were conducted with the approval of the Institutional Animal Care and Use Committee of Kyung Hee University, Korea [KHSASP-20-060].

### 2.5. Experimental Design

After 9 weeks of diabetes induction, 45 mice were divided into four groups as follows: (1) normal control group (CON; *n* = 11)—fed with a 10% kcal fat diet and supplemented with distilled water; (2) diabetes mellitus control group (DMC; *n* = 12)—fed with a 60% kcal fat diet and supplemented with distilled water; (3) low dosage of AME treated group (LAM; *n* = 11)—fed with the 60% kcal fat diet and supplemented with 50 mg/kg body weight (BW) of AME; (4) high dosage of AME treated group (HAM; *n* = 11)—fed with the 60% kcal fat diet and supplemented with 100 mg/kg BW of AME. AME was suspended in distilled water and orally gavaged every day for nine weeks. During the treatment period, body weight, amount of food intake, and 8 h-FBG levels were measured once a week.

At the end of the experiment, mice were anesthetized through inhalation of diethyl ether (Duksan, Ansan-si, Gyeonggi-do, Korea) and then blood was collected by cardiac puncture with heparinized syringe into an EDTA containing tube. Plasma was stored at −80 °C until analysis after isolated by centrifugation at 845× *g* for 15 min. The hepatic tissue was carefully separated, weighed, frozen in liquid nitrogen and kept at −80 °C for the following investigations. A portion of the liver was fixed in 10% formalin for histology analysis.

### 2.6. Oral Glucose Tolerance Test

The oral glucose tolerance test (OGTT) was performed at a week before sacrificing. After the overnight fasting, the mice were treated with 2 g/kg glucose (Sigma Aldrich, St. Louis, MO, USA) solution by oral gavage. Blood glucose levels were measured using One-touch select glucometer (LifeScan Inc., Milpitas, CA, USA) at 0 (baseline), 15, 30, 60, 90 and 120 min after glucose administration. The area-under-the-curve (AUC) was calculated by sum of the trapezoidal approximation for each mouse from 0 to 120 min. The AUC of each time point was determined using the following method:

OGTT AUC (mg/dL·min) = {15 min × (BG_0min_ + BG_15min_) × 1/2} + {15 min × (BG_15min_ + BG_30min_) × 1/2} + {30 min × (BG_30min_ + BG_60min_) × 1/2} + {30 min × (BG_60min_ + BG_90min_) × 1/2} + {30 min × (BG_90min_ + BG_120min_) × 1/2} (1)

### 2.7. Plasma Hemoglobin A1c (HbA1c) Level

Plasma HbA1c levels were measured using commercial reagent methods (Crystal Chem., Elk Grove Village, IL, USA).

### 2.8. Plasma Insulin Level

Plasma insulin levels were measured using ELISA commercial kits (RayBiotech, Inc., Norcross, GA, USA) according to the manufacturer’s instruction after the overnight fasting.

### 2.9. Hepatic Damage Related Markers

Hepatocellular injury was estimated by analyzing aspartate aminotransferase (AST) and alanine aminotransferase (ALT) concentrations from plasma. The analysis was performed using commercial kits (Asan pharmaceutical, Dongdaemun-gu, Seoul, Korea) according to the manufacturer’s instructions.

### 2.10. Hepatic Lipid Profiles

Triglyceride (TG) and total cholesterol (TC) in the liver were detected by using commercially manufactured assay kits (Asan pharmaceutical, Dongdaemun-gu, Seoul, Korea). Hepatic lipids were extracted by the Folch method (2:1 chloroform/methanol mixture) [29].

### 2.11. Histological Analysis

The liver tissues fixed in a 10% formalin solution were embedded in paraffin to observe the morphology. The embedded tissues were cut to a thickness of 4 μm each, and the process of deparaffinization and rehydration was performed while attached to the slide glass. The stained hepatic sections were observed using an optical microscope (Olympus Optical, Shinjuku-ku, Tokyo, Japan). The level of hepatic lipid accumulation was estimated using ImageJ software (U.S. National Institutes of Health, Bethesda, MD, USA).

### 2.12. Western Blot Assay

Extraction of cytoplasmic and nuclear protein in liver was performed by using the Nuclear/Cytosol Fractionation kit (BioVision, Milpitas, CA, USA) according to the manufacturer’s protocol after the tissue homogenized in phosphate-buffered saline (PBS). The quantity of protein was analyzed by bicinchoninic acid protein assay kit (BCA Protein Assay Kit; Thermo Fisher Scientific, Middlesex County, MA, USA).

The protein extract samples were separated by 6–15 percent of sodium dodecyl sulfate–polyacrylamide gel electrophoresis (SDS-PAGE) and transferred onto polyvinylidene-fluoride (PVDF) membranes (MilliporeSigma, Middlesex County, MA, USA). The membranes were blocked with 3% bovine serum albumin (BSA) and incubated with primary antibodies: SREBP1c, FAS, PPARα, p-Akt (Ser473), Akt, SIRT3, PGC1α, GLUT2, Nuclear factor erythroid-2-related factor 2 (Nrf2), PPARγ, Lamin B1 (Santa Cruz Biotechnology, CA, USA, 1:200), C/EBPα (Cell Signaling Technology, MA, USA, 1:2000), CPT1A, NAD(P)H Quinone Dehydrogenase 1 (NQO1), protein carbonyls (Abcam, Cambridge, UK, 1:2000), LC3, p-AMPK (Thr 172), AMPK, p-mTOR, mTOR, IRβ, IRS-1 (Cell Signaling Technology, MA, USA, 1:2000), 4-HNE (BD Biosciences, NJ, USA, 1:500), and α-tublin (Sigma Aldrich, MO, USA, 1:4000).

After the incubation, the membranes were washed with PBS-Tween 20 (1.059 g/mL) (PBS-T) three times, incubated with secondary antibodies (Bio-rad, Contra Costa County, CA, USA, 1:4000) and then washed with PBS-T again. Protein bands expressed by the Enhanced Chemiluminescence reagent (ECL substrate; Bio-rad, Contra Costa County, CA, USA) were recorded and quantified using a G box system (Syngene, Nuffield Road, Cambridge, UK).

### 2.13. Statistical Analysis

Data were presented as mean ± standard error of mean (SEM). The significance of differences among the groups was evaluated by one-way analysis of variance (ANOVA) according to Duncan’s multiple range test for each biomarker using an SPSS software (SPSS Inc., Chicago, IL, USA). A significance level of *p* < 0.05 was adopted.

## 3. Results

### 3.1. Identification of Major Natural Compounds in AME

**Figure 1:**
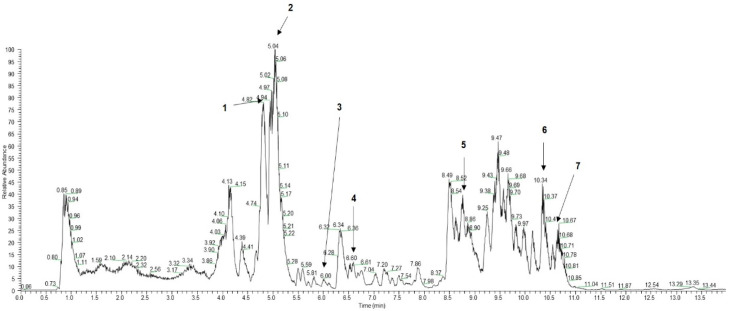
HPLC chromatogram of AME. (1) Rutin (Rt, 4.79), (2) Kaempferol-3-O-rutinoside (Rt, 5.04), (3) Quercetin (Rt, 6.03), (4) Kaempferol (Rt, 6.55), (5) Muricoreacin (Rt, 8.74), (6) Annonacin (Rt, 10.39) (7) Annonacinone (Rt, 10.59).

The present study indicated that AME exhibits antioxidants and hypoglycemic properties. Interestingly, acetogenins, rutin, quercetin, and kaempferol are known to have antioxidant effect and are major phytoconstituents of AME. HPLC was analyzed to verify the presence of these compounds in AME. HPLC analysis demonstrated that AME contains rutin, kaempferol-3-*O*-rutinoside, quercetin, kaempferol, muricoreacin, annonacin, and annonacinone (Figure 1).

### 3.2. The Total Phenolic Content

In AME, the total phenolic content was found to be 80.07 mg GAE/g of extract.

### 3.3. Effect of AME on Body Weight, Liver Weight and Diet Intake in Type 2 Diabetes Mice

There was a significant difference in body weight between the normal control mice and the type 2 diabetic mice during the experiment due to different type of diet treatment. On the other hand, the AME administered groups did not show any change on body weight when compared to the DMC group. In the liver tissue weight corrected by body weight, there was no significant difference between the CON group and the DMC group. Dietary intake was no statistical different among all groups (Table 1).

### 3.4. Effect of AME on Fasting Blood Glucose and Hemoglobin A1c in Type 2 Diabetes Mice

**Figure 2:**
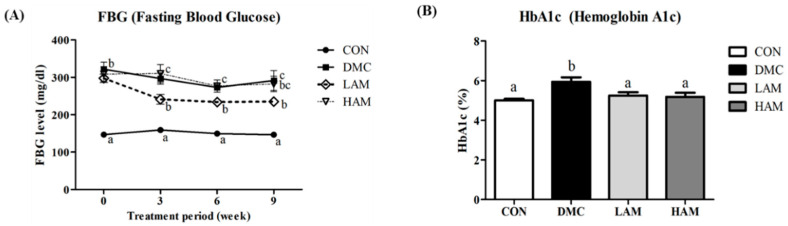
Effects of AME on (**A**) fasting blood glucose and (**B**) hemoglobin A1c (HbA1c) levels in T2DM mice All values are reported as mean and SEM. Values with the different superscript letter were significantly different (*p* < 0.05; ANOVA with post-hoc Duncan’s multiple range test). *n* = 10~11 mice in each group CON, normal control group (negative control); DMC, type 2 diabetic control group (positive control); LAM, diabetic group supplemented with low dosage (50 mg/kg BW) of AME; HAM, diabetic group supplemented with high dosage (100 mg/kg BW) of AME.

The fasting blood glucose (FBG) levels in the diabetic groups were significantly higher than that of the CON group. After three weeks of AME administration, the LAM group showed significantly lower blood sugar levels compared to the DMC group. This statistical difference was maintained until the end of the experiment (Figure 2A).

The level of glycated hemoglobin in the DMC group was significantly increased when compared to that of the CON group. On the other hand, hemoglobin A1c (HbA1c) levels were decreased in both the LAM and the HAM groups compared to those of the DMC group due to the effect of AME administration (Figure 2B).

### 3.5. Effect of AME on Glucose Intolerance and Insulin Resistance in Type 2 Diabetes Mice

**Figure 3:**
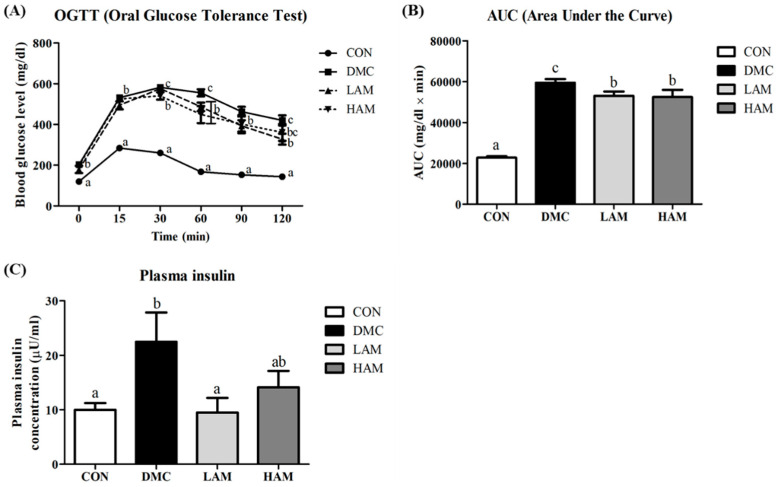
Effect of AME on glucose intolerance and insulin resistance, (**A**) OGTT, (**B**) OGTT AUC, and (**C**) plasma insulinT2DM mice All values are reported as mean and SEM. Values with the different superscript letter were significantly different (*p* < 0.05; ANOVA with post-hoc Duncan’s multiple range test). *n* = 10~11 mice in each group CON, normal control group (negative control); DMC, type 2 diabetic control group (positive control); LAM, diabetic group supplemented with low dosage (50 mg/kg BW) of AME; HAM, diabetic group supplemented with high dosage (100 mg/kg BW) of AME.

In FBG levels measured during OGTT progression, the diabetic group showed significantly higher values than the CON group. In particular, there was a significant difference in blood glucose levels at 30 and 60 min after oral glucose administration among the extract-supplemented group (both the LAM group and HAM group) and the DMC group (Figure 3A). As a result of the AUC measurement, the LAM group and the HAM group showed significantly lower values than the DMC group (Figure 3B). Plasma insulin level was significantly decreased in the LAM group than that of the DMC group (Figure 3C).

### 3.6. Effect of AME on Hepatic Damage in Type 2 Diabetes Mice

**Figure 4:**
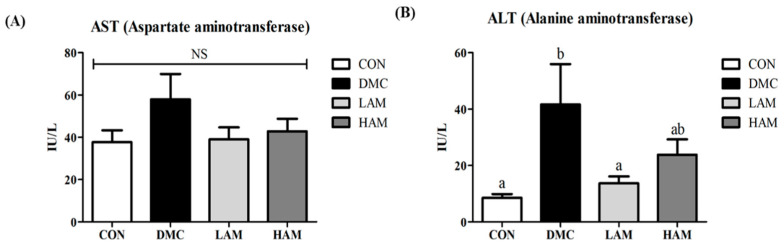
Effect of AME on hepatic damage related markers, (**A**) aspartate aminotransferase (AST) and (**B**) alanine aminotransferase (ALT) in T2DM mice All values are reported as mean and SEM. Values with the different superscript letter were significantly different (*p* < 0.05; ANOVA with post-hoc Duncan’s multiple range test). *n* = 10~11 mice in each group CON, normal control group (negative control); DMC, type 2 diabetic control group (positive control); LAM, diabetic group supplemented with low dosage (50 mg/kg BW) of AME; HAM, diabetic group supplemented with high dosage (100 mg/kg BW) of AME.

There was no significant difference in aspartate aminotransferase (AST) activity among all groups (Figure 4A). The level of alanine aminotransferase (ALT) was significantly higher in the DMC group than in the CON group. Low concentrations of AME supplementation significantly reduced ALT level. However, there was no difference in ALT level between the HAM group and the DMC group (Figure 4B).

### 3.7. Effect of AME on Hepatic Morphology and Hepatic Triglyceride (TG) and Total Cholesterol (TC) in Type 2 Diabetes Mice

**Figure 5:**
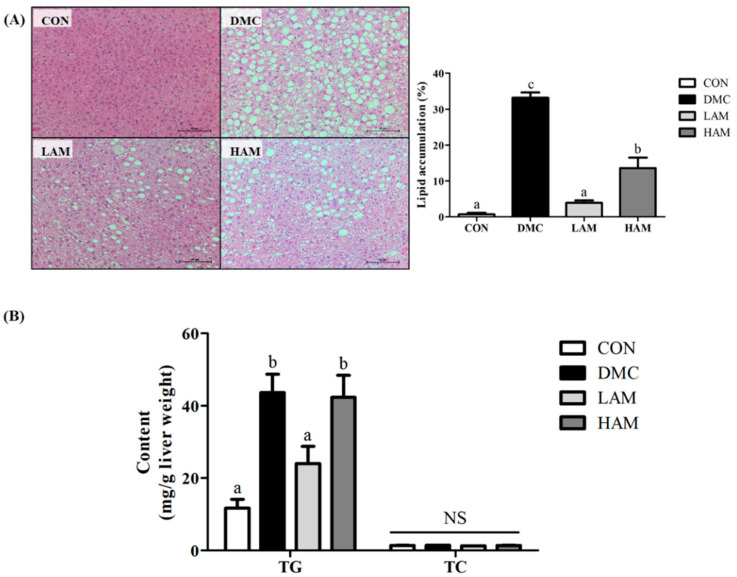
Effect of AME on hepatic (**A**) morphology (×200) and (**B**) hepatic TG and TC levels in T2DM mice All values are reported as mean and SEM. Values with the different superscript letter were significantly different (*p* < 0.05; ANOVA with post-hoc Duncan’s multiple range test). *n* = 4 mice in each group CON, normal control group (negative control); DMC, type 2 diabetic control group (positive control); LAM, diabetic group supplemented with low dosage (50 mg/kg BW) of AME; HAM, diabetic group supplemented with high dosage (100 mg/kg BW) of AME.

In representative histological micrographs of H&E-stained liver tissues, the DMC group showed markedly more fat deposition compared to the CON group. On the other hand, lipid droplets were decreased in the AME administration groups compared to that in the DMC group. Moreover, the size of the lipid droplet in the LAM group was also reduced compared to that in the DMC group, as shown in Figure 5A.

In the DMC group, hepatic TG content was abnormally higher in contrast to that of the CON group. However, it was reduced to almost normal level in the LAM group. On the other hand, hepatic TC content was not statistically different among all groups (Figure 5B).

### 3.8. Effects of AME on Insulin Signaling in Type 2 Diabetes Mice

**Figure 6:**
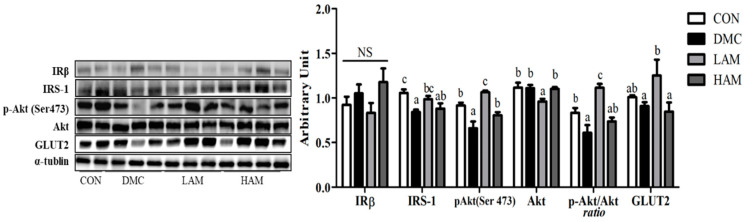
Effect of AME on insulin signaling in T2DM mice All values are reported as mean and SEM. Values with the different superscript letter were significantly different (*p* < 0.05; ANOVA with post-hoc Duncan’s multiple range test). *n* = 6 mice in each group CON, normal control group (negative control); DMC, type 2 diabetic control group (positive control); LAM, diabetic group supplemented with low dosage (50 mg/kg BW) of AME; HAM, diabetic group supplemented with high dosage (100 mg/kg BW) of AME.

IRS-1 level was significantly reduced in the DMC group compared to that of the CON group. At LAM group, IRS-1 level was significantly increased compared to the DMC group. On the other hand, as a result of measuring IRβ, there was no significant difference among all groups. There was a significant difference in the level of GLUT2 protein in hepatic tissue between the LAM group and the DMC group. Therefore, low concentration of AME supplementation enhanced the insulin signaling pathway through significant increases in IRS-1 and GLUT2 proteins compared to those of the DMC group. Akt phosphorylation showed a significant difference with a unique increase in the LAM group compared to that in the DMC group (Figure 6).

### 3.9. Effects of AME on Hepatic Oxidative Stress in Type 2 Diabetes Mice

**Figure 7:**
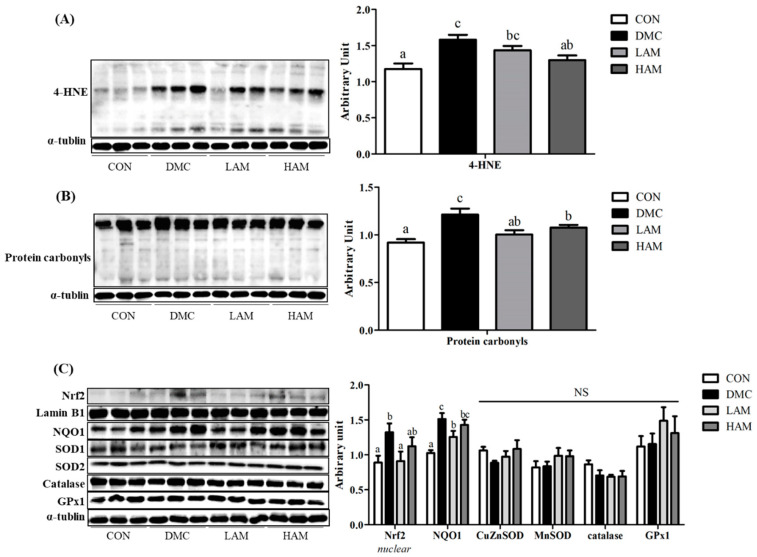
Effect of AME on (**A**) 4-HNE, (**B**) protein carbonyls, and (**C**) oxidative stress related markers in T2DM mice All values are reported as mean and SEM. Values with the different superscript letter were significantly different (*p* < 0.05; ANOVA with post-hoc Duncan’s multiple range test). *n* = 6 mice in each group CON, normal control group (negative control); DMC, type 2 diabetic control group (positive control); LAM, diabetic group supplemented with low dosage (50 mg/kg BW) of AME; HAM, diabetic group supplemented with high dosage (100 mg/kg BW) of AME.

Oxidative stress-related markers such as 4-HNE and protein carbonyls were measured in hepatic tissues. Both levels of 4-HNE and protein carbonyls were significantly increased in the DMC group compared to those of the CON group. In the HAM group, the level of 4-HNE was significantly reduced compared to that of the DMC group (Figure 7A).

In the LAM group, however, the levels of protein carbonyls but not the level of 4-HNE was significantly decreased compared to that of the DMC group (Figure 7B).

In this regard, the anti-oxidant effect of AME was also seen in the reduction of other oxidative stress related factors including Nrf2 and antioxidant enzymes. Nrf2 and NQO1 levels showed significant differences between the DMC and the LAM groups. On the other hand, there was no significant difference in the levels of oxidative stress related enzymes among all groups (Figure 7C).

### 3.10. Effects of AME on Energy Metabolism in Type 2 Diabetes Mice

**Figure 8:**
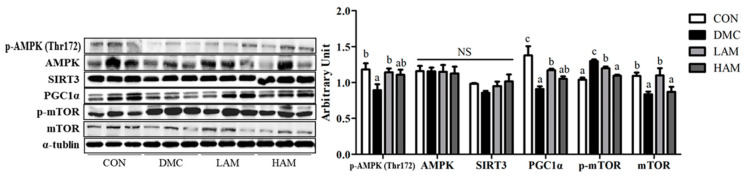
Effect of AME on energy metabolism in T2DM mice All values are reported as mean and SEM. Values with the different superscript letter were significantly different (*p* < 0.05; ANOVA with post-hoc Duncan’s multiple range test). *n* = 6 mice in each group CON, normal control group (negative control); DMC, type 2 diabetic control group (positive control); LAM, diabetic group supplemented with low dosage (50 mg/kg BW) of AME; HAM, diabetic group supplemented with high dosage (100 mg/kg BW) of AME.

As shown in Figure 8, energy metabolism related markers demonstrated by the AMPK—mTOR pathway in the DMC group were significantly reduced compared to those of the CON group. However, AME administration at a low dose but not at a high dose normalized energy metabolism through increased levels of p-AMPK and PGC1α. Further, p-mTOR was significantly decreased in the treatment groups compared to that of the DMC group.

### 3.11. Effects of AME on Autophagy in Type 2 Diabetes Mice

**Figure 9:**
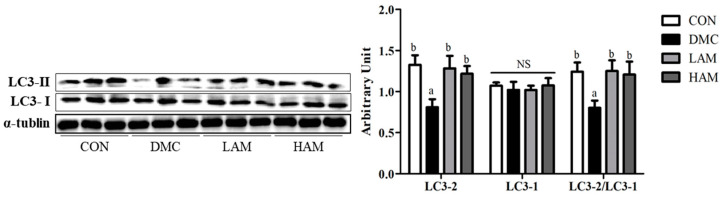
Effect of AME on autophagy in T2DM mice All values are reported as mean and SEM. Values with the different superscript letter were significantly different (*p* < 0.05; ANOVA with post-hoc Duncan’s multiple range test). *n* = 6 mice in each group CON, normal control group (negative control); DMC, type 2 diabetic control group (positive control); LAM, diabetic group supplemented with low dosage (50 mg/kg BW) of AME; HAM, diabetic group supplemented with high dosage (100 mg/kg BW) of AME.

The level of LC3-II expression in liver tissue was significantly lower in the DMC group than that in the CON group. In the AME treatment group regardless of dose, the LC3-II level was increased, showing a significant difference from that of the DMC group. On the other hand, there was no significant difference in the protein level of LC3-I among all groups. Therefore, the ratio of LC3-II/LC3-I showed a similar tendency to the level of LC3-II expression in all groups (Figure 9).

### 3.12. Effects of AME on Lipid Metabolism in Type 2 Diabetes Mice

**Figure 10:**
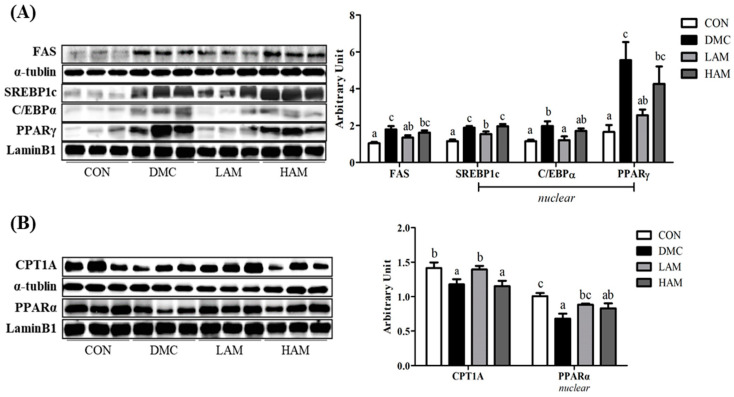
Effect of AME on lipid metabolism, (**A**) lipogenesis related markers (**B**) β-oxidation related markers, in T2DM mice All values are reported as mean and SEM. Values with the different superscript letter were significantly different (*p* < 0.05; ANOVA with post-hoc Duncan’s multiple range test). *n* = 6 mice in each group CON, normal control group (negative control); DMC, type 2 diabetic control group (positive control); LAM, diabetic group supplemented with low dosage (50 mg/kg BW) of AME; HAM, diabetic group supplemented with high dosage (100 mg/kg BW) of AME.

Proteins related to fat synthesis (FAS, SREBP1c, C/EBPα, PPARγ) were significantly induced in the DMC group compared to those of the CON group. Surprisingly, the levels of the proteins in the LAM group were reduced to normal levels when compared to those of the DMC group. On the other hand, the HAM group did not show any significant effect on all proteins related to fat synthesis (Figure 10A).

Hepatic fatty acid β-oxidation related markers in the DMC group were reduced compared to those of the CON group. Further, it was increased in the LAM group demonstrated by upregulation of PPARα—CPT1A pathway (Figure 10B).

## 4. Discussion

*Annona muricata* (AM), commonly known as graviola and soursop, is an evergreen plant of the *Annonaceae* family. The extract of *Annona muricata* is rich in bioactive compounds, such as acetogenins, flavonoids, tannins, alkaloids and, coumarins [22]. It is extensively used as a traditional medicine as an anti-hypertensive, anti-diabetic, anti-obesity, and anti-cancer agent. As shown in Figure 1, AM extract (AME) contains rutin, kaempferol-3-O-rutinoside, quercetin, quercitrin, kaempferol, muricoreacin, annonacin, and annonacinone. Furthermore, total polyphenol contents in AME (80.07 mg GAE/g) was higher than that of cherry tomato (13.51 mg GAE/g), apple peels (39 mg GAE/g), and *Actinidia chinensis* (Kiwifruit) (50.1 mg GAE/g) which are known to have a high antioxidant capacity [29,30,31]. These natural compounds showed antioxidant effects and exerted anti-diabetic and anti-lipogenic potentials in many studies [22,23,24].

The current study demonstrated that AME has a potential alleviating effect on diabetic fatty liver through protection of hepatic damage. Specifically, the results demonstrated that AME improved insulin metabolism, lipid metabolism, and autophagy pathway which are related with non-alcoholic fatty liver disease (NAFLD) risk in type 2 diabetes mellitus (T2DM).

T2DM is characterized by insulin resistance, in which the body usually does not fully respond to insulin [32]. Accordingly, the pathophysiology of T2DM is an abnormal increase in plasma glucose due to increased insulin resistance [33]. This study demonstrated that the administration of AME effectively regulated glucose homeostasis demonstrated by reduced fasting blood glucose level and plasma insulin level at low dose and hemoglobin A1c (HbA1c) and oral glucose tolerance test area-under-the-curve measurement at both doses in T2DM conditions. In previous studies, administration of the AME significantly attenuated blood glucose levels [22,25]. A recent study showed a similar tendency that rutin significantly reduced blood glucose and plasma insulin level in the hyperglycemic condition [34]. Furthermore, quercetin and kaempferol have been shown to decrease blood glucose level by regulation of energy homeostasis [35,36].

At the molecular level, a low dose of AME administration stimulated hepatic insulin signaling related proteins such as insulin receptor subunit (IRS)-1, glucose transporter (GLUT)2, and p-Akt compared to the DMC group. A previous study also showed that kaempferol supplementation alleviates insulin resistance via hepatic IκB kinase β (IKK)/nuclear factor kappa-β (NF-κB) signal in T2DM rats [37]. Therefore, administration of AME containing rutin, quercetin, kaempferol, acetogenins seems to be effective in diabetic metabolic abnormalities by attenuating blood sugar, reducing glucose intolerance, and enhancing insulin signaling pathway. The present study suggests that especially low dose of AME showed glycemic control effect on T2DM mice.

In T2DM condition, one of the major metabolic disorders due to insulin resistance is hyperlipidemia. Chronic hyperlipidemia in T2DM leads to an abnormal hepatic lipid metabolism and causes excess fat to accumulate, ultimately leading to NAFLD [38]. In previous studies, AME supplementation alleviated dyslipidemia in T2DM, especially through lowering hepatic triglycerides and plasma LDL cholesterol levels [25,38,39]. In accordance with the previous studies, this study demonstrated that, low dose of AME supplementation significantly reduced the hepatic TG concentration in the T2DM mice. Excess TG accumulation in hepatic tissue in diabetes is related to imbalance between lipogenesis and β-oxidation causing abnormal energy metabolism.

The key factor of energy metabolism regulation is 5’-AMP-activated protein kinase (AMPK), which can replace the NAD+/NADH ratio, thereby stimulating sirtuin (SIRT)1 expression in hepatocytes [40]. SIRT1 activation also increases fatty acid oxidation through peroxisome proliferator-activated receptor-α (PPARα) and peroxisome proliferator-activated receptor-γ coactivator 1-α (PGC1α), as well as decreases the inflammatory response through NFκB [41]. In this study, the LAM group significantly increased the levels of phosphorylated AMPK expression along with PGC1α in the diabetic mice. It might be inferred that AME administration contributes to normalization of energy metabolism through enhancement of AMPK/PGC1α levels, which were reduced in T2DM.

In addition, abnormal decline of AMPK causes overexpression of lipogenesis factors and continuous inhibition of β-oxidation. In particular, phosphorylation of AMPK increases the expression of sterol response element binding protein 1c (SREBP1c), thereby increasing the activity of factors such as fatty acid synthase (FAS), CCAAT-enhancer-binding proteins-α (C/EBPα), peroxisome proliferator-activated receptor-γ (PPARγ), and acetyl-CoA carboxylase (ACC) [42]. Furthermore, it inhibits the activity of factors such as carnitine palmitoyltransferase 1A (CPT1A) and peroxisome proliferator-activated receptor-α (PPARα), so that fat synthesis can be achieved more effectively. Accordingly, the de novo lipogenesis (DNL) process rapidly increases but followed by fat accumulation. In our investigation, the DNL activity of the DMC group was increased through an excess of fat synthesis related factors (FAS, SREBP1c, C/EBPα, PPARγ) and inhibition of β-oxidation related factors (CPT1A, PPARα). A previous study demonstrated that quercetin improved NAFLD by ameliorating lipid metabolism via AMPK/PPAR signaling pathway in *db*/*db* mice [43]. Furthermore, kaempferol and rutin exhibited hepatoprotective effects in a mouse model of NAFLD by facilitating fatty acid metabolism and inhibiting lipogenesis [44,45]. Surprisingly, low dose of AME supplementation ameliorative abnormal lipid metabolism under diabetic condition.

On the other hand, autophagy also plays a role in lipid metabolism homeostasis in hepatic tissues [46]. Autophagy is a critical system that maintains cellular homeostasis by inducing the death of cells that have expired or are abnormal. AMPK-mTOR signaling is organically linked to the level of LC3 expression which induces formation and maturation of autophagosomes. An abnormal autophagy caused liver disease by accelerating hepatic fat accumulation in the end stage of diabetes [16]. In the result of this study, autophagy pathway was inhibited by a decrease in p-AMPK and an increase in p-mTOR, thereby decreased LC3-2 level. As a result, prolonged synthetic action and low grade of lipophagy lead to the excess lipid accumulation inside the liver. However, autophagy was normalized in the LAM group through the enhanced AMPK with decreased p-mTOR level. The results suggest that AME supplementation could control hepatic lipid homeostasis along with lipophagy activation and AMPK-mTOR pathway in diabetic condition.

Accordingly, the hepatic lipid accumulation was reduced as shown in the histological analysis. As a result of measuring ALT, an index of hepatic damage, the LAM group showed significantly lower levels than the DMC group whereas high dose of AME could not reduce it. This shows that administration of AME protected hepatic damage through relief of fat accumulation. This result suggests that the protective effect of AME has not been shown to be dose-dependent and is likely to alleviate the incidence of diabetic liver disease in the long term.

Moreover, AME treatment reduced hyperglycemia induced hepatic oxidative stress. Oxidative stress refers to an imbalance between free radicals and antioxidants and causes damage to cells and tissues [47]. In response to oxidative stress, nuclear factor erythrocyte-related factor 2 (Nrf2) trigger expression of NAD(P)H quinone dehydrogenase 1 (NQO1) as a downstream protein for activation antioxidant enzymes. Increased levels of oxidation products such as 4-hydroxynonena (4-HNE) and protein carbonyls present cellular damage due to oxidative stress. AME lowered oxidative stress as shown by reduction of 4-HNE and protein carbonyls in the diabetic mice. Furthermore, the protein levels of Nrf2 and its down-regulation of NQO1 in the LAM group were decreased than that of the DMC group. It indicates that administration of AME has a potential to alleviate diabetic tissue damage through reduction of oxidative stress.

Collectively, our study found that low dose of AME treatment was more effective in regulation of insulin signaling, lipid metabolism, and energy metabolism compared to high dose of AME. Although a high dose of AME supplementation could not regulate some biomarkers (body and liver weight, plasma insulin, and hepatic TG level) showed similar effects on some biomarkers (HbA1c, AUC, hepatic lipid accumulation, pAkt, LC3, and protein carbonyls) as shown in low dose of AME treated group. In addition, the high dose of AME treated group were even more effective on some other biomarkers (4-HNE and pmTOR) compared to the low dose of AME treated mice. Furthermore, our unpublished data showed that high dose of AME supplementation had more beneficial effects on energy metabolism in other organs in T2DM mice. It can be considered that molecular pathways can be selectively regulated at different doses of AME and that the most effective dose of AME treatment might be tissue-specific in diabetes.

Thus, this current study suggested that AME, particularly at low doses, can be beneficial on glycemic control and hepatic lipid metabolism without dose dependent manner in hyperglycemia induced hepatic damage.

In the aspect of clinical applications, conversion of low dose of AME from mice to humans was calculated as 4.1 mg/kg. Although there might be the potentiality of the AME on diabetic patients, more studies are needed to consider the application of AME to humans with a careful approach as only male mice were used in this study.

## 5. Conclusions

This study demonstrated that AME treatment attenuated diabetic liver damage via the regulation of lipid homeostasis in T2DM. AME ameliorated insulin resistance through blood glucose lowering effect and insulin signaling stimulation. Furthermore, AME reduced hepatic oxidative stress induced by glucotoxicity and lipotoxicity under diabetic condition. AME also contributed to improving lipid catabolic reactions, such as β-oxidation and lipophagy, instead of de novo lipogenesis in diabetic liver. Taken together, AME could be helpful to establish preventive effects on NAFLD induced by hyperglycemia in type 2 diabetes.

## Figures and Tables

**Table 1:** Effect of AME on body weight, liver weight and diet intake in T2DM mice.

	CON	DMC	LAM	HAM
Body weight (g)				
before treatment	26.91 ± 0.61 ^a^	33.77 ± 1.56 ^b^	33.46 ± 0.92 ^b^	34.07 ± 1.30 ^b^
after treatment	30.71 ± 0.79 ^a^	38.67 ± 2.02 ^b^	38.49 ± 1.29 ^b^	40.23 ± 1.26 ^b^
change	3.80 ± 0.33 ^a^	4.90 ± 0.65 ^ab^	5.02 ± 0.76 ^ab^	6.16 ± 0.59 ^b^
Liver weight (% BW)	3.55 ± 0.08 ^a^	4.15 ± 0.21 ^ab^	3.78 ± 0.26 ^a^	4.63 ± 0.41 ^b^
Food intake (g/day)	2.72 ± 0.09	2.53 ± 0.11	2.68 ± 0.09	2.66 ± 0.09

All values are reported as mean and SEM. Values with the different superscript letter were significantly different (*p* < 0.05; ANOVA with post-hoc Duncan’s multiple range test). *n* = 10~11 mice in each group. CON, normal control group (negative control); DMC, type 2 diabetic control group (positive control); LAM, diabetic group supplemented with low dosage (50 mg/kg BW) of AME; HAM, diabetic group supplemented with high dosage (100 mg/kg BW) of AME.

## Data Availability

Data is contained within the article.
